# Effects of exercise interventions on cancer-related fatigue and quality of life among cancer patients: a meta-analysis

**DOI:** 10.1186/s12912-023-01363-0

**Published:** 2023-06-13

**Authors:** Xiaoli Chen, Juejin Li, Chongcheng Chen, Yalin Zhang, Shu Zhang, Yun Zhang, Lin Zhou, Xiaolin Hu

**Affiliations:** 1 Department of Nursing, West China Hospital, Sichuan University/West China School of Nursing, Sichuan University, Chengdu, PR China; 2 Department of Nephrology, West China Hospital, Sichuan University/West China School of Nursing, Sichuan University, Chengdu, PR China

**Keywords:** Cancer, Exercise, Cancer-related fatigue, Quality of life, Meta-analysis

## Abstract

**Purpose:**

In this study, exercise interventions were evaluated for their effects on cancer-related fatigue (CRF) and quality of life (QoL) among cancer patients.

**Design:**

A meta-analysis was performed.

**Methods:**

We systematically searched the PubMed/Medline, Web of Science, Embase, Cochrane Central Register of Controlled Trials (CENTRAL), PsycINFO, and CINAHL databases, and gray literature sources including the Virginia Henderson International Nursing Library and Google Scholar. This study only included randomized controlled trials (RCTs) examining how exercise interventions affect CRF and QoL among cancer patients. Based on the Cochrane Risk-of-Bias Assessment Tool, version 2 (RoB 2) and the Grading of Recommendations Assessment, Development and Evaluation (GRADE) approach, the methodological quality of the included studies was evaluated. In addition, standardized mean differences (SMDs) and 95% confidence intervals (CIs) were applied to assess the intervention effect with respect to CRF and QoL. Data analysis was performed using Review Manager (version 5.4).

**Results:**

There were a total of 1573 participants in the 28 included articles. According to the meta-analysis, CRF (SMD = -0.35, 95% CI: -0.63 to -0.07, p = 0.01) and QoL (SMD = 0.36, 95% CI: 0.20 to 0.53, p < 0.01) were positively affected by exercise interventions. Subgroup analyses revealed considerable improvements in CRF (SMD = -0.54, 95% CI: -1.00 to -0.09, p = 0.02) and QoL (SMD = 0.38, 95% CI: 0.16 to 0.59, p < 0.01) from aerobic exercise. An intervention duration less than 12 weeks had a better effect on CRF (SMD = -0.80, 95% CI: -1.43 to -0.17, p = 0.01) and QoL (SMD = 0.53, 95% CI: 0.21 to 0.85, p < 0.01), and three times per week was the most effective frequency in improving QoL (SMD = 0.69, 95% CI: 0.28 to 1.11, p < 0.01). Exercise intervention was more successful in improving CRF (SMD = -0.66, 95% CI: -1.10 to -0.21, p < 0.01) and QoL (SMD=-0.50, 95% CI: 0.23 to 0.78, p < 0.01) in female cancer patients. Sensitivity analyses showed that the pooled outcomes were reliable and stable.

**Conclusion:**

Exercise interventions are a workable approach to improve CRF and QoL among cancer patients. An aerobic exercise intervention of less than 12 weeks might be most effective in improving CRF and QoL, and three times per week might be the most appropriate frequency. Exercise might have a more positive effect on improving CRF and QoL in female cancer patients. Additionally, a larger number of high-quality RCTs should be conducted to further confirm the efficacy of exercise interventions on CRF and QoL among cancer patients.

**Registration number:**

CRD42022351137.

**Supplementary Information:**

The online version contains supplementary material available at 10.1186/s12912-023-01363-0.

## Introduction

The World Health Organization states that the incidence and mortality rates of cancer are increasing rapidly worldwide, making it the world’s second leading cause of death [1]. The International Agency for Research on Cancer predicts that 30.2 million cases of cancer will be diagnosed worldwide by 2040, a 36.1% increase from 2020 (19.3 million) [2]. With improvements in cancer diagnosis and treatment approaches, the survival years of cancer patients have greatly increased [3]. However, their QoL has not significantly improved [3]. Specifically, cancer patients’ QoL is markedly affected for up to 2 to 26 years after cancer diagnosis with the influence of a variety of problems [4, 5], among which CRF is one of the most common causes [6, 7].

CRF is a multidimensional, persistent, and painful feeling experienced in relation to cancer and its treatment, which is described as a prolonged debilitating condition and interferes with cancer patients’ body functions and their daily activities [8–10]. CRF is one of the most common symptoms observed among cancer patients, and its incidence during and after positive cancer treatment is 40% ~ 100% and 14% ~ 40%, respectively [11]. Notably, CRF is stubborn in cancer patients and can last up to five years or more during their survival phases and lead to substantial impairments in various aspects [12–14]. For example, CRF increases the physical and mental burden of cancer patients, resulting in physical dysfunctions such as pain and insomnia and psychological problems including anxiety and depression [15, 16], which can seriously impair their QoL [7, 17]. QoL for cancer patients refers to a dynamic and subjective feeling that involves all aspects of their lives and needs [18]. Poor QoL is associated with a series of adverse events, such as aggravating physical dysfunction and delaying cancer rehabilitation, leading to an increase in symptom burden, including persistent CRF, and a decrease in survival rate [19–21]. Thus, there is an urgency to implement effective and sustainable interventions to address CRF and QoL among cancer patients. Many studies have reported that non-pharmacological interventions could improve CRF and QoL [22, 23]. These non-pharmacological interventions do not lead to any serious adverse side-effects and are free from drug interactions, which makes them easier to accept by cancer patients than drug treatments [24, 25].

Exercise interventions are one of the most effective non-pharmacological interventions to address the side effects of treatment, with the aims of improving physical fitness and helping symptom management in a systematic and scientific manner [26–28]. In recent years, exercise interventions have been widely conducted among patients diagnosed with various chronic diseases, including chronic obstructive pulmonary disease, high blood pressure, and diabetes, and have been considered effective for protecting the heart, strengthening body immunity, as well as improving fatigue and QoL [29–31]. Some studies have implemented exercise interventions in cancer care and have revealed benefits for cancer patients from both physical (e.g., improving sleep) and mental (e.g., reducing psychological distress) aspects [32, 33].

Nevertheless, studies on exercise interventions for cancer patients remain limited, and the majority of these studies have targeted patients with breast and lung cancer [34, 35]. Current studies have revealed the effectiveness of exercise interventions for cancer patients’ physical health, including pain and insomnia, and mental health, including anxiety and depression [36, 37]. However, the impact on CRF and QoL is controversial. Results from previous randomized controlled trials have shown that exercise fails to improve CRF and QoL [38, 39], whereas some studies have reported that exercise is effective for CRF and QoL [40, 41]. Hence, further studies are needed. Moreover, to the best of our knowledge, few studies have conducted systematic and quantitative assessments of CRF and QoL among cancer patients with respect to the type, duration, and frequency of exercise interventions, as well as the gender of cancer patients. Thus, there is a need to perform further quantitative synthesis. The purposes of this meta-analysis were (1) to determine the overall effect of exercise interventions on CRF and QoL among patients with cancer and (2) to identify the effects of different exercise types, durations, and frequencies, as well as cancer patient gender on CRF and QoL.

## Methods

This meta-analysis was registered in the International Prospective Register of Systematic Reviews (CRD42022351137), and the Preferred Reporting Items for Systematic Reviews and Meta-Analyses (PRISMA) guidelines [42] were implemented for reporting. Since the data of this review were obtained from formerly published studies, there was no requirement for informed consent or ethical permission.

### Data sources and search strategies

We searched the PubMed/Medline, Web of Science, Cochrane Central Register of Controlled Trials (CENTRAL), PsycINFO, CINAHL, and Embase electronic databases to find relevant articles up to 31 October 2022. In addition, searching of the gray literature was conducted, including the Virginia Henderson International Nursing Library and Google Scholar. A combination of MeSH terms and free-text terms was used. In the Supplementary Materials, we provided a detailed description of the search strategy (see **Supplementary file** Table 1s), which focuses solely on the study of humans and adults. The list of references to relevant articles was examined to find additional articles.

**Table 1:** Characteristics of included studies

Study (first author, year, country)	Number of participants at baseline / at postintervention(n)	Participants of characteristics			Exercise group: Delivery model; Type of exercise program; Frequency; Length of exercise bout	Control group	Intervention duration
		Mean Age (SD) (year)	Type of cancer	Stage of cancer			
A.Murtezani, 2014 Kosovo	73 / 62	EG: 53.00(11.00); CG: 51.00(11.00)	Breast cancer	I - III	Supervised; Aerobic; 3 times per week; NA	Usual care	10-week
B.R. Villumsen, 2019 Denmark	46 / 41	EG: 67.60(4.60); CG: 69.80(4.40)	Prostate cancer	Locally advanced or advanced stage disease	Home-based; Mixed; 4 times per week; 90 min	Usual care; each patient kept a diary of his self-reported exercise	12-week
C.C.Henke, 2014 Germany	43 / 29	older than 18 years	Lung cancer patients (non-small cell lung cancer or small cell lung cancer	III - IV	Supervised; Mixed; The endurance training:5 days a week/The strength training: every other day of the week; NA	Conventional care; only conventional physiotherapy	64-week
C.J.Taso, 2014 China	60 / 60	Total: 49.27(10.23)	Nonmetastatic breast cancer	I - III	NA; Mixed; 2 times per week; 60 min	Standard care; maintained an ordinary daily activity routine	8-week
C.L.Hwang, 2012 China	24 / 24	EG: 61.00(6.30); CG: 58.50(8.20)	Non-small cell lung cancer	III - IV	Supervised; Aerobic; 3 times per week; 30–40 min	Usual care, general patient education, and social phone calls every 2–3 weeks, without supervised exercise intervention	8-week
C.M. Donnelly, 2011 UK	33 / 29	EG: 53.50(8.70); CG: 52.10(11.80)	Gynaecological cancer survivors	I - III	Home-based; Mixed; 5 days per week; 30 min	Standard care; weekly telephone calls for the duration of the intervention	12-week
C.Saraboon, 2021 Thailand	30 / 26	EG: 45.07 (3.88); CG: 45.53 (4.64)	NA	No reported	Supervised; Mixed; 2 times per week; 60 min	Conventional care	6-week
D.Reis, 2013 USA	41 / 29	EG: 54.00(11.10); CG: 59.00(10.70)	Breast cancer	I - III	NA; Mixed; 3 times per week; 20-60 min	Usual care,continue normal activities	12-week
D.W.Kang, 2022 Canada	52 / 50	Total: 63.40(7.10)	Prostate cancer	No reported	Supervised; Aerobic; 3 times per week; NA	Usual care	12-week
F.Shobeiri, 2016 Iran	60 / 53	EG: 42.70(9.60); CG: 43.50(8.60)	Breast cancer	I - II	Supervised; Aerobic; 2 times per week; 40-60 min	Usual care	10-week
J.F. Christensen, 2019 Denmark	39 / 34	EG: 57.80(10.40); CG: 60.30 (8.90)	Colorectal cancer	I - III	Home-based; Aerobic; At least 1 per 3 weeks; 150 min	Usual care	12-week
J.H.Hwang, 2008 Korea	40 / 37	EG: 46.30(7.50); CG: 46.30(9.50)	Breast cancer	No reported	Supervised; Mixed; 3 times per week; 50 min	Self-shoulder stretching exercise	5-week
J.M.Broderick, 2013 Ireland	43 / 40	EG: 52.30(8.30); CG: 51.20(10.30)	All types of cancer	I - III	Supervised; Aerobic; 2 times per week; 45 min	Usual care; maintain habitual levels of activity	8-week
J.Y. Kim, 2019 Korea	71 / 58	Total: 56.80(10.20)	Colorectal cancer	II - III	Home-based; Aerobic and resistance exercises; the first 6 weeks:18 metabolic equivalent of task (MET) hours per week/After 6 weeks:27 MET-hours per week; 30 min	Usual care; continue with their usual activities or exercises	12-week
K.A.Nyrop, 2017 USA	78 / 62	age 21 or older	Breast cancer	I - IV	NA; Aerobic; at least 150 min per week; NA	Wait List Control	6-week
K.Gokal, 2016 UK	50 / 50	EG: 52.08(11.70); CG: 52.36(8.90)	Breast cancer	I - III	Home-based; Aerobic; 5 times per week; 30 min	Usual care	12-week
K.S. Courneya 2003 Canada	53 / 52	EG: 59.00(5.00); CG: 58.00(6.00)	Postmenopausal Breast Cancer	I - III	Supervised; Resistance; 3 times per week; began at 15 min for weeks 1 through 3, and then systematically increased by 5 min every 3 weeks thereafter to 35 min for weeks 13 through 15	Usual care; Usual care; Not training and not being asked to start an structured exercise program	15-week
K.S.Courneya, 2007-AET Canada	158 / 128	mean 49 years	Breast cancer	I - II	Supervised; Aerobic; 3 times per week; 60 min	Usual care; do not start an exercise training program during chemotherapy	24-week
K.S.Courneya, 2007-RET Canada	162 / 133	mean 49 years	Breast cancer	I - II	Supervised; Resistance; 3 times per week; two sets of 8 to 12 repetitions	Usual care; do not start an exercise training program during chemotherapy	24-week
K.S.Courneya, 2009 Canada	122 / 117	EG: 52.80(14.75); CG: 53.50(15.5)	Lymphoma	I - IV	Supervised; Aerobic; 3 times per week; Duration began 15 to 20 min for the first 4weeks and increased by 5 min per week to 40 to 45 min in the ninth week	Usual care; no increase in exercise	12-week
L.Bourke, 2018 UK	50 / 46	EG: 68.00 (6.00); CG: 67.00 (9.00)	Localised prostate Cancer	I - II	Supervised; Aerobic; 150 min per week; NA	Usual care	48-week
M.L.McNeely, 2008 Canada	52 / 52	EG: 53.00(11.00); CG: 57.00(8.25)	Head and Neck Cancer	I - IV	Supervised; Mixed; a minimum of 2 times per week; NA	A standardized therapeutic exercise protocol (TP)(the current standard of care)	12-week
S.B.Santagnello, 2020 Brazil	26 / 20	EG: 52.10(10.10); CG: 59.00(9.20)	Breast cancer	I - III	Supervised; Resistance; 3 times per week on non-consecutive days; between 8 and 12 repetitions per set	Only stretching exercises twice a week	12-week
S.C.Adams, 2018 Canada	63 / 62	EG: 44.00(11.60), CG: 43.30(9.90)	Testicular cancer survivors	I - IV	Supervised; Mixed; 3 times per week; 35 min	Usual care	12-week
S.Cataldi, 2019 Italy	20 / 20	EG: 53.30(19.20); CG: 52.00(15.70)	All types of cancer	No reported	Supervised; Resistance; 2 times per week; 60 min	Wait List Control	8-week
S.Mijwel, 2019 Sweden	132 / 117	EG: 54.40(10.30); CG: 52.60(10.20)	Breast cancer	I - III	Supervised; Aerobic; 2 times per week; 60 min	Usual care	16-week
T.R.S.Paulo, 2019 Brazil	36 / 29	EG: 63.20(7.10); CG: 66.60(9.60)	Older breast cancer	I - III	Supervised; Aerobic and resistance exercises; 3 times per week; 40 min	Participate in stretching and relaxation exercises, 2 times per week, with 45 min session durations, for 9 months, and the exercises were active, lasting 10–15 s each	36-week
W.Ndjavera, 2020 UK	50 / 50	EG: 71.40(5.40); CG: 72.50(4.20)	Prostate cancer	Locally advanced、Metastatic	Supervised; Aerobic and resistance exercises; 2 times per week; 60 min	Usual care; did not receive any supervised exercise or specific physical activity recommendations	12-week
W.Zhou, 2018 China	114 / 83	18 year ≤ age ≤ 70 yr	Nasopharyngeal carcinoma	III - IV	Supervised; Aerobic; 5 times per week; 60 min	Usual care	80-week

Abbreviations: SD = standard deviation, EG = exercise group, CG = control group, NA = not available, AET = aerobic exercise training, RET = resistance exercise training

### Inclusion and exclusion criteria

The studies were independently screened and selected by two investigators based on the following inclusion criteria: (1) the study participants were patients aged over 18 years and diagnosed with any type of cancer, regardless of sex and cancer stage; (2) the intervention group used exercise interventions only; (3) the comparators of the studies were routine care, usual care, wait list control, standard treatment, or conventional care interventions; (4) the outcomes of the studies were CRF and QoL; and (5) the studies were in English or Chinese. The exclusion criteria were as follows: (1) the studies were research protocols, conference abstracts, reviews, systematic evaluations, meta-analyses, pilot research, or duplicate reports; and (2) the studies failed to record available data. There were no restrictions in terms of publication dates.

### Study selection and data extraction

Data management was enabled by the reference management program Endnote X9. After removal of duplicates, two authors individually filtered all titles and abstracts that met the eligibility criteria. Then, the full texts of any citations deemed possibly related by either author were retrieved and assessed. We resolved disagreements through discussion or through consultation with third authors as necessary.

The relevant data were extracted from the included studies by means of a predesigned data gathering form. Extracted data covered the study’s first author, date of publication, country, sample size, characteristics of participants (mean age, cancer type and stage), intervention and control type, and intervention duration.

### Quality assessment

Included studies were independently evaluated for methodological quality by two authors applying the Cochrane Risk-of-Bias Assessment Tool, version 2 (RoB2) [43], which included five domains: randomization process, deviations from intended interventions, missing outcome data, measurement of the outcome, and selection of the reporting results. Within each field, the studies were evaluated as having a low risk of bias, some concerns, or high risk of bias. If disagreement occurred, the reviewers reached a consensus, with a third reviewer resolving disagreements or discussing them within the team, if needed.

The evidence quality was evaluated using the Grading of Recommendations Assessment, Development and Evaluation (GRADE) approach [44] according to the following domains: risk of bias, inconsistency, indirectness, imprecision, and publication bias. The total classification of the evidence was assessed as “very low”, “low”, “moderate” or “high”. Methodologically, the evidence quality for RCTs was originally classified as high, with a reduction to moderate, low or very low if limitations were detected in any of the above domains. Nonetheless, the evidence could be escalated through a dose‒response gradient and a large effect. Two researchers independently scored evidence for quality in accordance with the GRADE manual. Disagreements were settled with discussion or with a third author’s assistance.

### Data synthesis and analysis

Data synthesis and analysis in this study were carried out using Review Manager 5.4 software. Means and standard deviations (SDs) at post-intervention were extracted for meta-analysis. Due to the different measurements used in these enrolled studies, standardized mean differences (SMDs) and 95% confidence intervals (CIs) were used to evaluate the intervention effect with respect to CRF and QoL. A two-tailed p value < 0.05 was applied to denote statistical significance. The I^2^ statistic and p value were used to assess heterogeneity. If I^2^ ≤ 50% and p > 0.1, the heterogeneity was considered statistically significant and was aggregated by a fixed effects model. If I^2^ > 50% and p < 0.1, a random effects model was used. Sensitivity analyses were performed to examine the stability of the pooled outcomes, and meta-regression analyses were conducted for exploring potential sources of heterogeneity. Subgroup analyses based on intervention type, duration, and frequency, as well as gender of cancer patient were also conducted in a predesigned manner. Funnel plots were examined to assess potential publication bias for CRF and QoL. Additionally, if the included study reported more than two arms, the method of splitting shared groups was used [45].

## Results

### Study selection

**Fig. 1:**
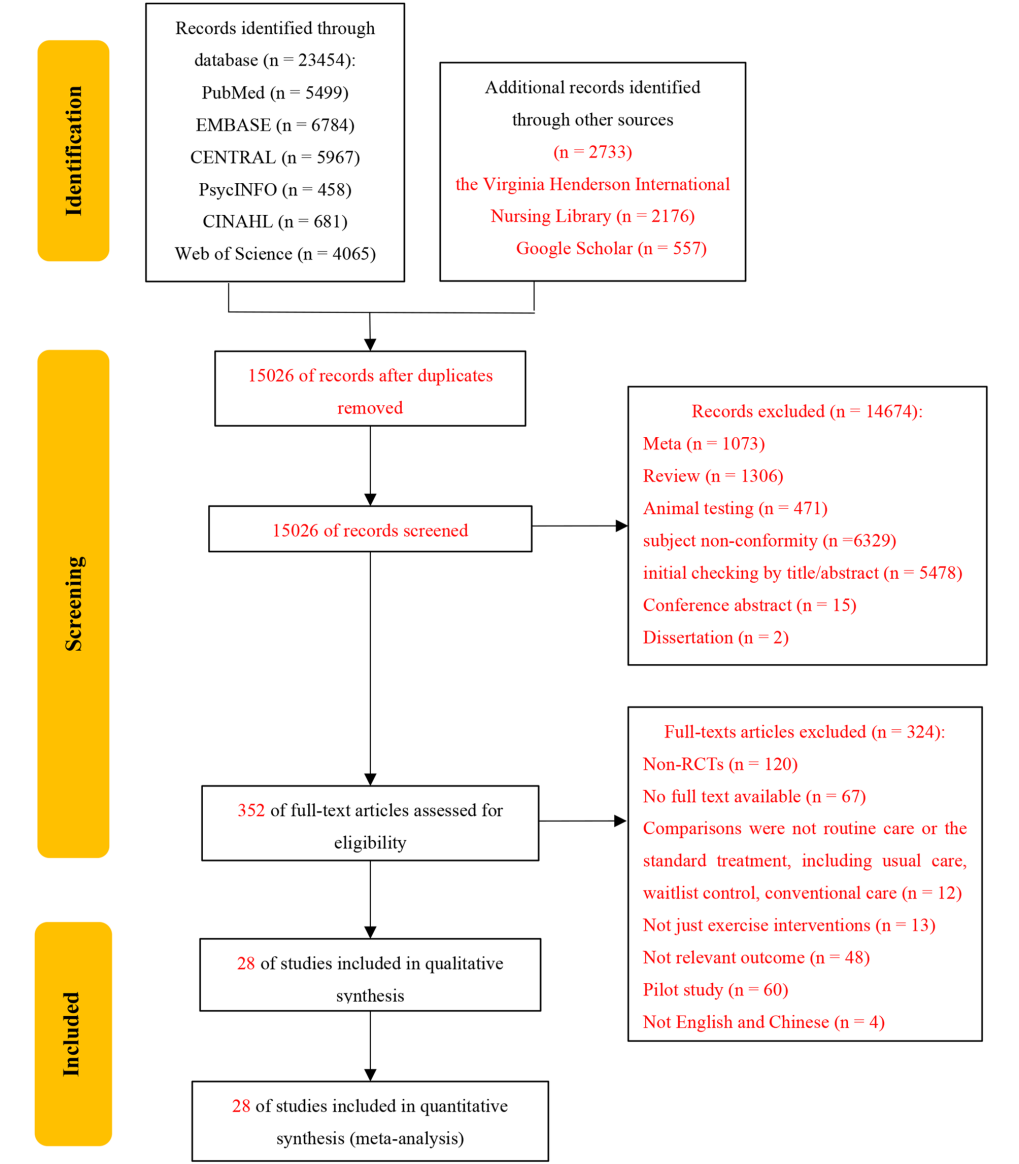
Flowchart of study selection and literature screening process

The selection process of our study is illustrated in Fig. 1. In brief, 26,187 references were retrieved from the databases; these were reduced to 352 after excluding references due to duplicate papers (11,161 studies), meta-analyses (1073 studies), reviews (1306 studies), animal testing studies (471 studies), subject nonconformity (6329 studies), initial checking by title/abstract (5478 studies), conference abstracts (15 studies), and dissertations (2 studies). A full-text review and quality assessment of the remaining 352 studies was performed. After full-text review, 324 studies were excluded for the reasons outlined in Fig. 1. Finally, a total of 28 RCTs [38–41, 46–69] were subjected to data extraction.

### Characteristics of the included studies

#### Study characteristics

Across studies, sample sizes ranged from 20 to 133, totalling 1573 samples. In the intervention group, the mean age (SD) ranged from 42.70 (9.60) to 71.40 (5.40) years, while in the control group, it ranged from 43.50 (8.60) to 72.50 (4.20) years. Among the 28 studies, 13 included only breast cancer survivors, four included prostate cancer patients, three included lung cancer patients, and two included colorectal cancer patients. And 24 studies reported the cancer stages of the participants. One study was a 3-arm RCT. The studies were published from 2003 to 2022, and their characteristics are shown in Table 1.

#### Characteristics of exercise interventions

Most exercise interventions were supervised or home-based. The types of exercise interventions varied across studies and included walking, cycling, Tai Chi, yoga, and jogging. Specifically, 14 studies [41, 46, 48, 50, 51, 53, 55–58, 60, 65–67] evaluated aerobic exercise, four studies [47, 49, 50, 63] evaluated resistance exercise including recumbent or upright cycle ergometers, leg extensions, and leg curls, three studies [40, 59, 61] evaluated aerobic and resistance exercise, and eight studies [38, 39, 52, 54, 62, 64, 68, 69] evaluated mixed exercise. And 12 studies reported an intervention duration < 12 weeks, and 20 studies reported an intervention duration ≥ 12 weeks. In addition, exercise interventions ranged in frequency from two to five times per week (n = 24), four to five days per week (n = 1), 150 min per week (n = 2), endurance training five days per week and strength training every other day (n = 1), and 18 metabolic equivalent task (MET) hours per week for the first six weeks and 27 MET hours per week after six weeks (n = 1).

#### Characteristics of comparators

Within the control groups, 23 studies used usual care/conventional care/standard treatment or standard care, two studies used wait list controls, three studies provided participants with simple stretching and/or relaxation exercises.

#### Outcome measurements

Among these included studies, the outcome assessment tools differed. In terms of CRF, three studies applied the European Organization for Research and Treatment of Cancer QoL Questionnaire Core 30 (EORTC-QLQ-C30), five studies applied the Functional Assessment of Cancer Therapy-Fatigue (FACT-F), and three studies applied the Functional Assessment of Cancer Therapy-Anemia (FACT-An) scales. Regarding QoL, seven studies applied the Functional Assessment of Cancer Therapy scale (FACT), and three of the seven applied the Functional Assessment of Cancer Therapy-General (FACT-G) scale.

### Risk of bias

**Fig. 2:**
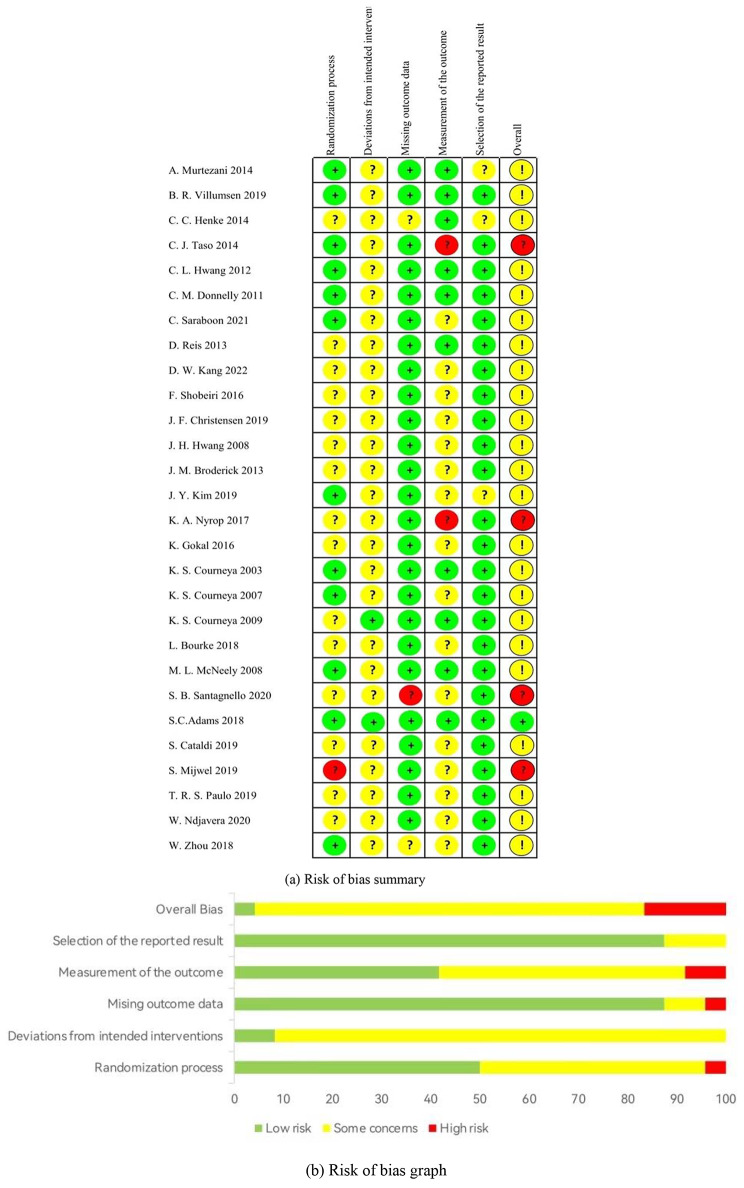
The results of risk of bias assessment of included studies

For each study, RoB 2 was applied to evaluate the risk of bias, and the outcomes are displayed in Fig. 2. Specifically, although all included studies were reported as randomized, selective bias remained, as 15 studies did not mention allocation concealment. All studies were evaluated as having some concerns regarding deviations from intended intervention because no blind method on participants was reported. Additionally, one study reported incomplete data, resulting in a high risk of attrition bias. Regarding bias in outcome measures, ten studies were blinded to the outcome assessor, 16 studies were concerned, and the other two studies were at high risk. In addition, three studies presented inadequate information to make judgments, which may result in effects being overvalued and a selection bias in reported results being introduced. Overall, one study was assessed as “low-risk bias”, twenty-three studies were classified as “some concern” and four studies were evaluated as “high risk of bias”. The funnel plots were distributed roughly symmetrically, with no apparent differences (see Figure S1a and Figure S1b of Supplementary file).

### Quality of evidence

The evidence certainty for CRF and QoL was rated as low and high, respectively. The total sample size for all two study indicators was greater than 300. The results of the evaluation and further detailed information are displayed in Table 2.

**Table 2:** GRADE evidence profile

Outcomes	Certainty assessment						Effect				Certainty
	Risk of bias	Inconsistency	Indirectness	Imprecision	Others		No. of Studies	No. of Individuals	Rate (95%CI)		
Cancer-related fatigue	Serious^a^	Very serious^b^	Not serious^c^	Not serious^d^	Strong association^e^		22RCTs	1197	SMD − 0.35 SD (− 0.63 to − 0.07)		⨁⨁◯◯ Low
Quality of life	Serious^a^	Not serious^f^	Not serious^c^	Not serious^d^	Strong association^e^		12RCTs	590	SMD 0.36 SD (0.20 to 0.53)		⨁⨁⨁⨁ High

Abbreviations: CI: confidence intervals; RCT = Randomized controlled trial; SMD: standardized mean difference; SD: standard deviation

Notes: a. most of the included studies were assessed as some concerns/high-risk bias

b. I^2^ > 75%

c. direct participants, interventions and outcomes

d. total sample size > 300

e. The entire 95% CI does not contain 0

f. I^2^ < 50%

### Meta-analysis results

#### Effect of exercise interventions on CRF

**Fig. 3:**
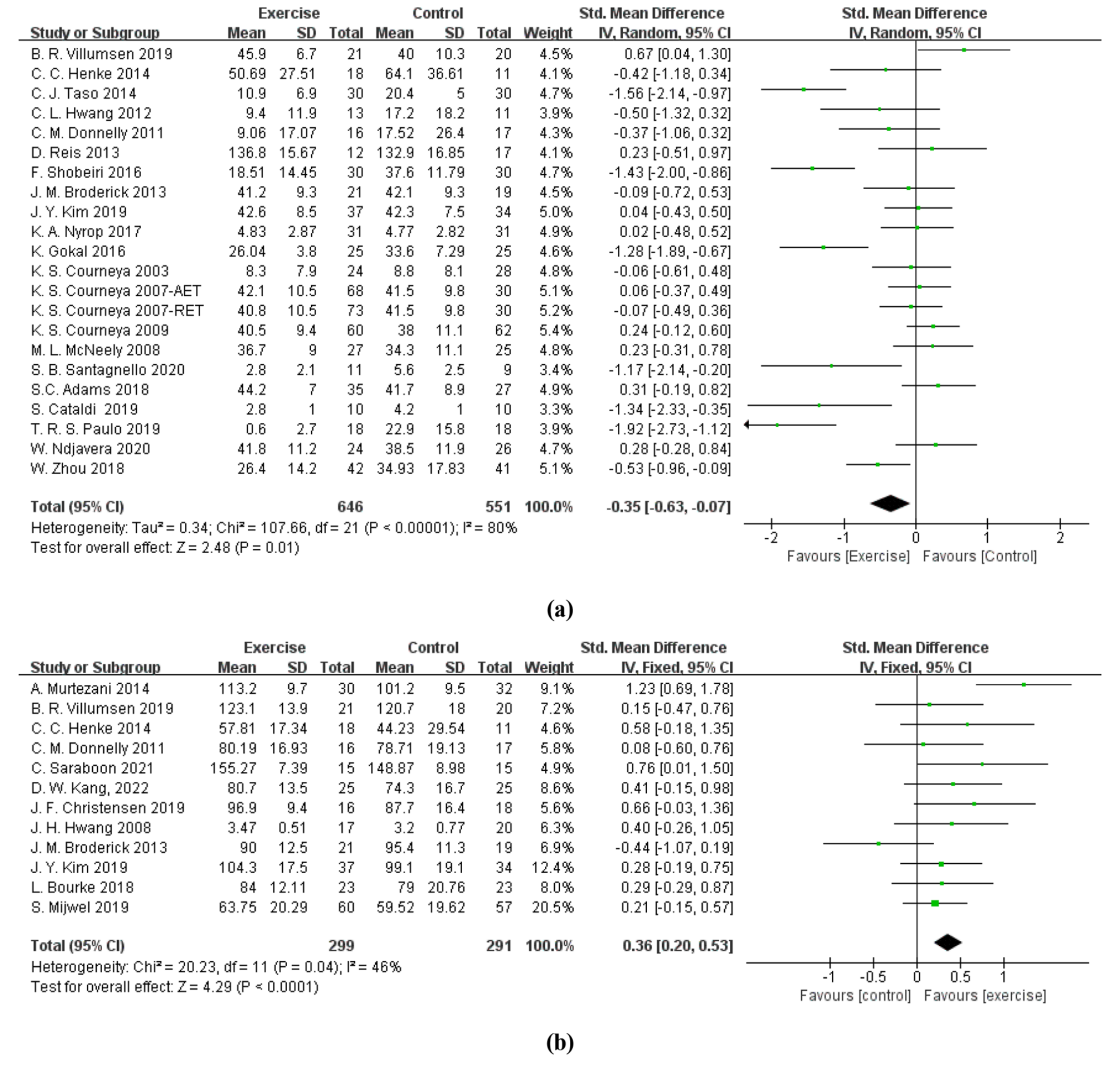
Forest plots of the total effect of exercise intervention on **(a)** cancer-related fatigue and **(b)** quality of life. CI = confidence interval, SD = standard deviation, AET = aerobic exercise training, RET = resistance exercise training

Twenty-two studies [38–41, 47, 49–55, 59–63, 65–67] estimated the effects of exercise interventions on CRF among cancer patients. Exercise interventions significantly decreased CRF among cancer patients (SMD = -0.35, 95% CI: -0.63 to -0.07, p = 0.01; I^2^ = 80%, p < 0.01) (Fig. 3a). A sensitivity analysis was conducted with a leave-one-out approach, and the results were between − 0.28 (95% CI: -0.54 to -0.02) and − 0.40 (95% CI: -0.67 to -0.12).

Subgroup analyses based on exercise intervention type were conducted, including aerobic (SMD = -0.54, 95% CI: -1.00 to -0.09, p = 0.02), resistance (SMD = -0.52, 95% CI: -1.11 to 0.08, p = 0.09), aerobic and resistance (SMD = -0.49, 95% CI: -1.61 to 0.63, p = 0.39), and mixed exercise interventions (SMD = 0.15, 95% CI: -0.16 to 0.47, p = 0.35). Our results showed high heterogeneity in all subgroups (Figure S2a).

In the subgroup analyses, the duration of exercise interventions was evaluated, including less than 12 weeks (SMD = -0.80, 95% CI: -1.43 to -0.17, p = 0.01) and greater than or equal to 12 weeks (SMD = -0.18, 95% CI: -0.46 to 0.10, p = 0.20). High heterogeneity remained in these subgroups (Figure S3a).

Subgroup analyses were also conducted in accordance with the frequency of the exercise interventions. To ensure the accuracy of the results, some articles that did not report the frequency of exercise were excluded from this subgroup analysis [46, 51, 54]. Subgroup analyses included 2 times per week (SMD = -0.15, 95% CI: -1.03 to 0.73, p = 0.75), 3 times per week (SMD = -0.23, 95% CI: -0.60 to 0.15, p = 0.24), and more than 3 times per week (SMD = 0.39, 95% CI: -0.99 to 0.21, p = 0.20). Our results suggested high heterogeneity in all subgroups (Figure S4a).

For the gender of cancer patients, subgroup analyses excluded studies that did not report gender [38, 40, 41, 47, 51, 54, 55, 67] to secure the credibility of this study. Subgroup analysis included female (SMD = -0.66, 95% CI: -1.10 to -0.21, p < 0.01) and male (SMD = 0.40, 95% CI: 0.07 to 0.72, p = 0.02). Our outcomes showed that there was low heterogeneity in the male subgroup (I^2^ = 0%, P = 0.61) (Figure S5a).

#### Effect of exercise interventions on QoL

Twelve studies [39–41, 46, 48, 52, 54, 56–58, 64, 68] estimated the effects of exercise interventions on QoL among cancer patients. Exercise considerably improved the QoL of cancer patients (SMD = 0.36, 95% CI: 0.20 to 0.53, p < 0.01; I^2^ = 46%, p < 0.05) (Fig. 3b). A sensitivity analysis was carried out with a leave-one-out method, with results ranging from 0.27 (95% CI: 0.10 to 0.45) to 0.42 (95% CI: 0.25 to 0.59).

Subgroup analyses were conducted by the type of exercise intervention, including aerobic (SMD = 0.38, 95% CI: 0.16 to 0.59, p < 0.01) and mixed exercise (SMD = 0.34, 95% CI: 0.08 to 0.59, p = 0.01). Our results indicated that there was low heterogeneity for the subgroup of other interventions (I^2^ = 0%, p = 0.76) (Figure S2b).

Subgroup analyses on the duration of the exercise interventions were performed and included less than 12 weeks (SMD = 0.53, 95% CI: 0.21 to 0.85, p < 0.01) and greater than or equal to 12 weeks (SMD = 0.30, 95% CI: 0.10 to 0.49, p < 0.01). There was high heterogeneity in subgroups based on interventions less than 12 weeks (I^2^ = 81%, p < 0.01) (Figure S3b).

Regarding the frequency of exercise interventions, subgroup analyses removed studies [32, 46] that did not specify the frequency of exercise to ensure the credibility of this study. The subgroup analysis included 2 times per week (SMD = 0.15, 95% CI: -0.42 to 0.73, p = 0.60), 3 times per week (SMD = 0.69, 95% CI: 0.28 to 1.11, p < 0.01), and more than 3 times per week (SMD = 0.24, 95% CI: -0.15 to 0.63, p = 0.23). Our results suggested low subgroup heterogeneity for exercise interventions performed 3 times per week (I^2^ = 45%, p = 0.14) and more than 3 times per week (I^2^ = 0%, p = 0.59) (Figure S4b).

As for cancer patients’ gender, subgroup analyses eliminated studies that did not report gender [40, 41, 48, 52, 54, 64] to assure the credibility of this study. The subgroup analysis included female (SMD=-0.50, 95% CI: 0.23 to 0.78, p < 0.01) and male (SMD = 0.29, 95% CI: -0.05 to 0.63, p = 0.09). Our outcomes indicated a high heterogeneity in the female subgroup (I^2^ = 79%, p < 0.01) (Figure S5b).

### Results of meta-regression analyses

#### Results of meta-regression analysis of CRF

The results of the meta-regression included exercise types (R^2^ = 2.44%, p = 0.28), exercise duration (R^2^ = 0%, p = 0.43), exercise frequency (R^2^ = 0%, p = 0.52), gender (R^2^ = 0%, p = 0.62), and cancer types (R^2^ = 15.66%, p = 0.14). As a result, exercise type, duration, frequency, gender, and cancer type were not potential sources of heterogeneity (**see Supplementary file** Table 2s).

#### Results of meta-regression analysis of QoL

The results of the meta-regression included exercise types (R^2^ = 0%, p = 0.43), exercise duration (R^2^ = 0%, p = 0.33), exercise frequency (R^2^ = 0%, p = 0.75), gender (R^2^ = 0%, p = 0.40), and cancer types (R^2^ = 0%, p = 0.43). As a result, exercise type, duration, frequency, gender, and cancer type were not potential sources of heterogeneity (**see Supplementary file** Table 2s).

## Discussion

### Summary of main results

Our subgroup results showed that exercise duration of less than 12 weeks was effective in improving CRF, while greater than or equal to 12 weeks was invalid; regarding QoL, the effect of exercise duration less than 12 weeks was significantly superior to that of greater than or equal to 12 weeks. Exercise three times a week was effective for QoL, but not for CRF. Moreover, exercise intervention was effective in improving CRF and QoL in female cancer patients. The quality of evidence ranged from low to high. We explored possible sources of heterogeneity in this review in terms of the characteristics of the intervention (type of exercise, duration of exercise, and frequency of exercise) and participants (gender and different types of cancer), although none was statistically significant.

### Effectiveness of exercise interventions on CRF

The pooled results indicated that exercise interventions were effective for improving CRF among patients with cancer, which was in accordance with the results of previous studies [70, 71]. Exercise is generally considered an effective approach to improve dysfunction by increasing daytime activities, which can directly improve CRF [72, 73]. Owing to cancer diagnosis and treatment, cancer patients experience grievous CRF stemming from frequent cancer pain [74]. Regular physical exercise provides long-term benefits in reducing the intensity of cancer pain and its disruption of daily life [75], thereby assisting cancer patients in easing CRF. Furthermore, current evidence identified that CRF is secondary to immune dysregulation and inflammatory problems [16, 22]. One probable mechanism is that exercise interventions help cancer patients to strengthen the immune system, regulate body balance, and control inflammation, thereby alleviating fatigue [32, 76].

### Effectiveness of exercise interventions on QoL

Regarding QoL, the pooled results showed that exercise interventions were also beneficial in improving QoL among patients with cancer, which was in line with the results described in a prior meta-analysis [77]. One possible explanation is that exercise helps alleviate the symptom distress of cancer patients, which is a critical factor affecting their overall QoL [78]. For example, the release of dopamine from exercise can make patients feel happy, and thus, they can manage negative emotions effectively [79–81], and the reinforcement of the immune system can also improve their overall physical fitness [82]. In these ways, cancer patients’ QoL could be improved. Furthermore, unhealthy lifestyles (e.g., a sedentary lifestyle) following a cancer diagnosis could also impact cancer patients’ QoL [83]. Exercise has been demonstrated to be a cost-effective healthy lifestyle habit [84], and maintaining physical activity among cancer patients can benefit the self-management of their cancer-related symptoms. For instance, boosting their motivation to adopt more health-promoting actions and preventing the adverse effects associated with cancer treatment, such as pain and loss of muscle strength, provides them with ongoing benefits [85–87].

### Subgroup analysis

The results of our subgroup analysis of exercise types suggested that aerobic exercise is most effective for improving CRF and QoL among cancer patients, and the results were in agreement with the findings of previous studies [88, 89]. Cancer patients may tend to avoid exercise due to the burden of symptoms (such as CRF and pain) caused by cancer treatment [90]. Aerobic exercise seems more acceptable by cancer patients than other exercise types because it has lower intensity [91] and fewer potential adverse events, such as falls and muscle soreness [92]. Surprisingly, our results indicated that exercise interventions with durations shorter than 12 weeks showed better effectiveness for cancer patients than those with durations longer than 12 weeks, which was also confirmed in previous studies [93]. This might be because exercise interventions over long periods are likely to result in poor exercise program completion, as cancer patients may lack the motivation to persist [94]. Thus, developing the exercise habits of cancer patients may be a focus of future studies, and longer follow-ups are warranted to verify their exercise compliance and the long-term effects of exercise for patients with cancer.

Regarding the frequency of exercise, the results of our subgroup analysis revealed that exercising three times a week was most effective in enhancing QoL, which is compatible with the 2018 American College of Sports Medicine (ACSM) roundtable [95] stating that engaging in exercise three times a week improves QoL among cancer patients with additional benefits. One possible reason is that an exercise frequency of 3 times a week is easier for cancer patients to accommodate and follow and may also be more appropriate for their exercise tolerance [86], thus contributing to substantial improvements in their health-related well-being and QoL. However, only four studies in this meta-analysis reported that exercising three times a week significantly improved QoL; therefore, further studies with larger sample sizes are warranted. On the other hand, our results indicated that exercising three times per week did not alleviate CRF, which is not in line with the 2018 ACSM roundtable [95] results. CRF is among the most common adverse events during cancer treatment, and cancer patients may suffer from stubborn CRF, even with exercise interventions [96]. Frequent exercise interventions may cause severe postexercise discomfort in cancer patients, which may exacerbate CRF, especially during active cancer treatment [91, 97]. Therefore, further studies are needed to evaluate the viability and rationality of exercise interventions based on the specificity of CRF and to explore appropriate exercise frequencies that effectively improve CRF.

In addition, our subgroup results indicated that exercise greatly improved CRF and QoL in female cancer patients, while the effects were not significant in male. Previous studies have reported higher levels of CRF and worse QoL in females with cancer [98, 99], with the possibility that this may lead to a more significant effect of exercise interventions, from a statistical aspect. On the other hand, females with cancer have more acceptability and greater use of positive adaptive coping strategies, such as healthy behaviors like exercise, to face the disease compared to male cancer patients who tend to adopt an attitude of avoidance and denial [100–102]. Hence, female cancer patients may have higher adherence to exercise interventions, leading to better outcomes. Due to the small sample size, the results need to be treated with caution. Therefore, future studies could focus more on the effect of exercise on CRF and QoL in different gender cancer patients to validate our results.

### Strengths and limitations

This meta-analysis had several strengths: (a) to the best of our knowledge, this study is the first to examine how different types, durations and frequencies of exercise affect cancer patients’ CRF and QoL; (b) with only the RCTs included in this meta-analysis, there was an enhancement in the methodological quality of the study; (c) the searches were conducted in six major electronic databases by using a combination of MeSH terms and keywords covering cancer and exercise to minimize potential publication bias; and (d) our findings were reliable based on the sensitivity analysis. However, the present study also had some limitations: (a) the majority of studies suffered from a lack of blinding of participants and assessors; (b) due to the small number of relevant included studies, we were unable to perform subgroup analyses of cancer stage, thus failing to identify the heterogeneity of patient groups and the potential association with outcomes; and (c) despite a comprehensive search strategy, we were unable to uncover the full texts of some studies. Thus, our conclusions need to be interpreted with caution.

### Implications for nursing practice and further research

Based on the results of our study that we recommend aerobic exercise 3 times a week for cancer patients. Notably, our results suggested that short-term exercise interventions are more effective for cancer patients compared to long-term exercise. Thus, future studies should consider tailoring stage-specific exercise programs to cancer patients in different stages. Moreover, previous studies have shown the effectiveness of online exercise interventions [103], whereas the studies included in our meta-analysis were based entirely on offline activities. Hence, it is imperative to transfer exercise interventions from the real world to online against the background of the COVID-19 epidemic as a serious worldwide public health problem [104]. For example, an artificial intelligence exercise app for cancer patients led by medical staff and a social platform similar to WeChat can be developed to give cancer patients good health education and guidance to improve cancer-related symptoms and QoL. In addition, future studies with larger sample sizes are needed to focus on the impact of gender in the exercise of cancer patients.

## Conclusions

In brief, exercise interventions are effective in relieving CRF and improving QoL among patients with cancer. In this study, the most effective improvement in CRF and QoL was an aerobic exercise intervention of less than 12 weeks, and regarding the frequency of the intervention, three times a week was the most beneficial. Exercise intervention was more significant in improving CRF and QoL in female cancer patients. Moreover, stage-specific exercise interventions should be developed for patients with different cancer stages, and online factors should receive further attention and exploration and be introduced into future exercise intervention studies.

## Electronic supplementary material

Below is the link to the electronic supplementary material.

Supplementary Material 1

## Data Availability

All data generated or analyzed during this study is included in this published article.

## Abbreviations

CRF: Cancer-related fatigue
QoL: Quality of life
