# Supplementary material for: Effects of exercise interventions on cancer-related fatigue and quality of life among cancer patients: a meta-analysis
Source: BMC Nurs. 2023 Jun 13;22:200. doi: 10.1186/s12912-023-01363-0 (PMC10261838; doi:10.1186/s12912-023-01363-0)
Supplement: Supplementary file 1 — Supplementary Material 1 [file 12912_2023_1363_MOESM1_ESM.docx]

**Supplementary material**

**Table 1s.** Search strategy for each database.

| **Database** | **Search strategy** |
| --- | --- |
| PubMed | **(((("Neoplasms"[Mesh]) OR ((((((((((((((((((Tumor[Title/Abstract]) OR (Neoplasm[Title/Abstract])) OR (Tumors[Title/Abstract])) OR (Neoplasia[Title/Abstract])) OR (Neoplasias[Title/Abstract])) OR (Cancer[Title/Abstract])) OR (Cancers[Title/Abstract]))) OR (Malignant Neoplasm[Title/Abstract])) OR (Malignancy[Title/Abstract])) OR (Malignancies[Title/Abstract])) OR (Malignant Neoplasms[Title/Abstract])) OR (Neoplasm, Malignant[Title/Abstract])) OR (Neoplasms, Malignant[Title/Abstract])) OR (Benign Neoplasms[Title/Abstract])) OR (Benign Neopiasm[Title/Abstract])) OR (Neoplasms, Benign[Title/Abstract])) OR (Neoplasm,Benign[Title/Abstract]))) AND ("Exercise"[Mesh])) OR (((((((((((((((((((((((((Exercises[Title/Abstract]) OR (Physical Activity[Title/Abstract])) OR (Activities, Physical[Title/Abstract])) OR (Activity,Physical[Title/Abstract])) OR (Physical Activities[Title/Abstract])) OR (Exercise. Physical[Title/Abstract])) OR (Exercises.Physical[Title/Abstract])) OR (Physical Exercise[Title/Abstract])) OR (Physical Exercises[Title/Abstract])) OR (Acute Exercise[Title/Abstract])) OR (Acute Exercises[Title/Abstract])) OR (Exercise. Acute[Title/Abstract])) OR (Exercises,Acute[Title/Abstract])) OR (Exercise, Isometric[Title/Abstract])) OR (Exercises. isometric[Title/Abstract])) OR (Isometric Exercises[Title/Abstract])) OR (Isometric Exercise[Title/Abstract])) OR (Exercise, Aerobic[Title/Abstract])) OR (Aerobic Exercise[Title/Abstract])) OR (Aerobic Exercises[Title/Abstract])) OR (Exercises. Aerobic[Title/Abstract])) OR (Exercise Training[Title/Abstract])) OR (Exercise Trainings[Title/Abstract])) OR (Training. Exercise[Title/Abstract])) OR (Trainings,Exercise[Title/Abstract]))) AND (((Randomized controlled trial[Title/Abstract]) OR (RCT[Title/Abstract])) OR (Random*[Title/Abstract]))** |
| Embase | (Neoplasms or Tumor or Neoplasm or Tumors or Neoplasia or Neoplasias or Cancer or Cancers or Malignant Neoplasm or Malignancy or Malignancies or Malignant Neoplasms or Neoplasm, Malignant or Neoplasms, Malignant or Benign Neoplasms or Benign Neopiasm or Neoplasms, Benign or Neoplasm,Benign) and (Exercise or Exercises or Physical Activity or Activities, Physical or Activity,Physical or Physical Activities or Exercise. Physical or Exercises.Physical or Physical Exercise or Physical Exercises or Acute Exercise or Acute Exercises or Exercise. Acute or Exercises,Acute or Exercise, Isometric or Exercises. isometric or Isometric Exercises or Isometric Exercise or Exercise, Aerobic or Aerobic Exercise or Aerobic Exercises or Exercises. Aerobic or Exercise Training or Exercise Trainings or Training. Exercise or Trainings,Exercise) and (RCT or Randomized controlled trial or Random*)).tw |
| Cochrane Central Register of Controlled trials (CENTRAL) | **((Neoplasms or Tumor or Neoplasm or Tumors or Neoplasia or Neoplasias or Cancer or Cancers or Malignant Neoplasm or Malignancy or Malignancies or Malignant Neoplasms or Neoplasm,Malignant or Neoplasms,Malignant or Benign Neoplasms or Benign Neoplasm or Neoplasms, Benign or Neoplasm,Benign) and (Exercise or Exercises or Physical Activity or Activities, Physical or Activity,Physical or Physical Activities or Exercise,Physical or Exercises,Physical or Physical Exercise or Physical Exercises or Acute Exercise or Acute Exercises or Exercise,Acute or Exercises,Acute or Exercise, Isometric or Exercises, Isometric or Isometric Exercises or Isometric Exercise or Exercise, Aerobic or Aerobic Exercise or Aerobic Exercises or Exercises, Aerobic or Exercise Training or Exercise Trainings or Training, Exercise or Trainings, Exercise) and (RCT or Randomized controlled trial or Random*)).ab.** |
| *PsyCINFO* | **SU ( Neoplasms OR Tumor OR Neoplasm OR Tumors OR Neoplasia OR Neoplasias OR Cancer OR Cancers OR Malignant Neoplasm OR Malignancy OR Malignancies OR Malignant Neoplasms OR Neoplasm, Malignant OR Neoplasms, Malignant OR Benign Neoplasms OR Benign Neoplasm OR Neoplasms, Benign OR Neoplasm, Benign ) AND SU ( Exercise OR Exercises OR Physical Activity OR Activities, Physical OR Activity, Physical OR Physical Activities OR Exercise, Physical OR Exercises, Physical OR Physical Exercise OR Physical Exercises OR Acute Exercise OR Acute Exercises OR Exercise, Acute OR Exercises, Acute OR Exercise, Isometric OR Exercises, Isometric OR Isometric Exercises OR Isometric Exercise OR Exercise, Aerobic OR Aerobic Exercise OR Aerobic Exercises OR Exercises, Aerobic OR Exercise Training OR Exercise Trainings OR Training, Exercise OR Trainings, Exercise ) AND AB ( RCT OR Randomized controlled trial OR Random* )** |
| CINAHL | **TX(Neoplasms OR Tumor OR Neoplasm OR Tumors OR Neoplasia OR Neoplasias OR Cancer OR Cancers OR Malignant Neoplasm OR Malignancy OR Malignancies OR Malignant Neoplasms OR Neoplasm, Malignant OR Neoplasms, Malignant OR Benign Neoplasms OR Benign Neoplasm OR Neoplasms, Benign OR Neoplasm, Benign) AND TX(Exercise OR Exercises OR Physical Activity OR Activities, Physical OR Activity, Physical OR Physical Activities OR Exercise, Physical OR Exercises, Physical OR Physical Exercise OR Physical Exercises OR Acute Exercise OR Acute Exercises OR Exercise, Acute OR Exercises, Acute OR Exercise, Isometric OR Exercises, Isometric OR Isometric Exercises OR Isometric Exercise OR Exercise, Aerobic OR Aerobic Exercise OR Aerobic Exercises OR Exercises, Aerobic OR Exercise Training OR Exercise Trainings OR Training, Exercise OR Trainings, Exercise) AND AB(RCT OR Randomized controlled trial OR Random*)** |
| Web of science | **(TS=(Neoplasms) OR AB=(Tumor OR Neoplasm OR Tumors OR Neoplasia OR Neoplasias OR Cancer OR Cancers OR Malignant Neoplasm OR Malignancy OR Malignancies OR Malignant Neoplasms OR Neoplasm, Malignant OR Neoplasms, Malignant OR Benign Neoplasms OR Benign Neoplasm OR Neoplasms, Benign OR Neoplasm, Benign))AND (TS=(Exercise) OR AB=(Exercises OR Physical Activity OR Activities, Physical OR Activity, Physical OR Physical Activities OR Exercise, Physical OR Exercises, Physical OR Physical Exercise OR Physical Exercises OR Acute Exercise OR Acute Exercises OR Exercise, Acute OR Exercises, Acute OR Exercise, Isometric OR Exercises, Isometric OR Isometric Exercises OR Isometric Exercise OR Exercise, Aerobic OR Aerobic Exercise OR Aerobic Exercises OR Exercises, Aerobic OR Exercise Training OR Exercise Trainings OR Training, Exercise OR Trainings, Exercise)) AND( AB=(RCT OR Randomized controlled trial OR Random*))** |
| Google Scholar | **#allintitle: cancer and exercise "Randomized controlled trial"**  **#allintitle: cancer and physical activity "Randomized controlled trial"**  **#allintitle: cancer and activity "Randomized controlled trial"**  **#allintitle: Neoplasms and exercise "Randomized controlled trial"**  **#allintitle: Neoplasms and training "Randomized controlled trial"** |
| virginia henderson international nursing library | **(TS=(Neoplasms) OR AB=(Tumor OR Neoplasm OR Tumors OR Neoplasia OR Neoplasias OR Cancer OR Cancers OR Malignant Neoplasm OR Malignancy OR Malignancies OR Malignant Neoplasms OR Neoplasm, Malignant OR Neoplasms, Malignant OR Benign Neoplasms OR Benign Neoplasm OR Neoplasms, Benign OR Neoplasm, Benign))AND (TS=(Exercise) OR AB=(Exercises OR Physical Activity OR Activities, Physical OR Activity, Physical OR Physical Activities OR Exercise, Physical OR Exercises, Physical OR Physical Exercise OR Physical Exercises OR Acute Exercise OR Acute Exercises OR Exercise, Acute OR Exercises, Acute OR Exercise, Isometric OR Exercises, Isometric OR Isometric Exercises OR Isometric Exercise OR Exercise, Aerobic OR Aerobic Exercise OR Aerobic Exercises OR Exercises, Aerobic OR Exercise Training OR Exercise Trainings OR Training, Exercise OR Trainings, Exercise)) AND( AB=(RCT OR Randomized controlled trial OR Random*))** |

**Table 2s.** Results of potential sources of heterogeneity

| **Possible sources of heterogeneity** | **Cancer-related fatigue** | |  | **Quality of life** | |
| --- | --- | --- | --- | --- | --- |
|  | **R^2^ (amount of heterogeneity accounted for)** | **p** |  | **R^2^(amount of heterogeneity accounted for)** | **p** |
| Exercise types | 2.44% | 0.28 |  | 0.00% | 0.43 |
| Exercise duration | 0.00% | 0.43 |  | 0.00% | 0.33 |
| Exercise frequency | 0.00% | 0.52 |  | 0.00% | 0.75 |
| Gender | 0.00% | 0.62 |  | 0.00% | 0.40 |
| Cancer types | 15.66% | 0.14 |  | 0.00% | 0.43 |

**
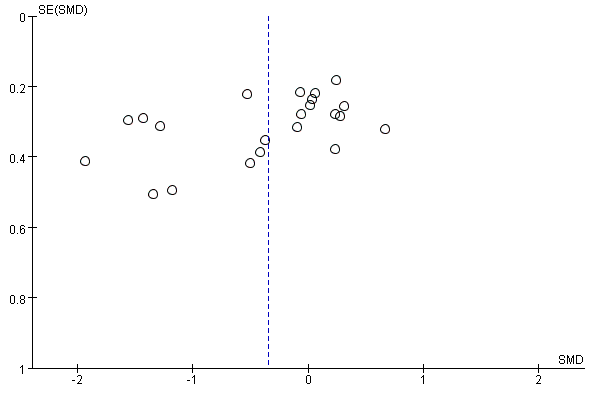
**

**(a)**

**
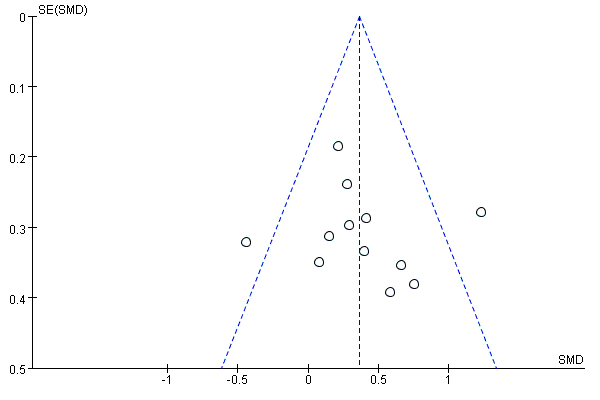
**

**(b)**

**Figure S1.** Funnel plot of (a) cancer-related fatigue and (b) quality of life.


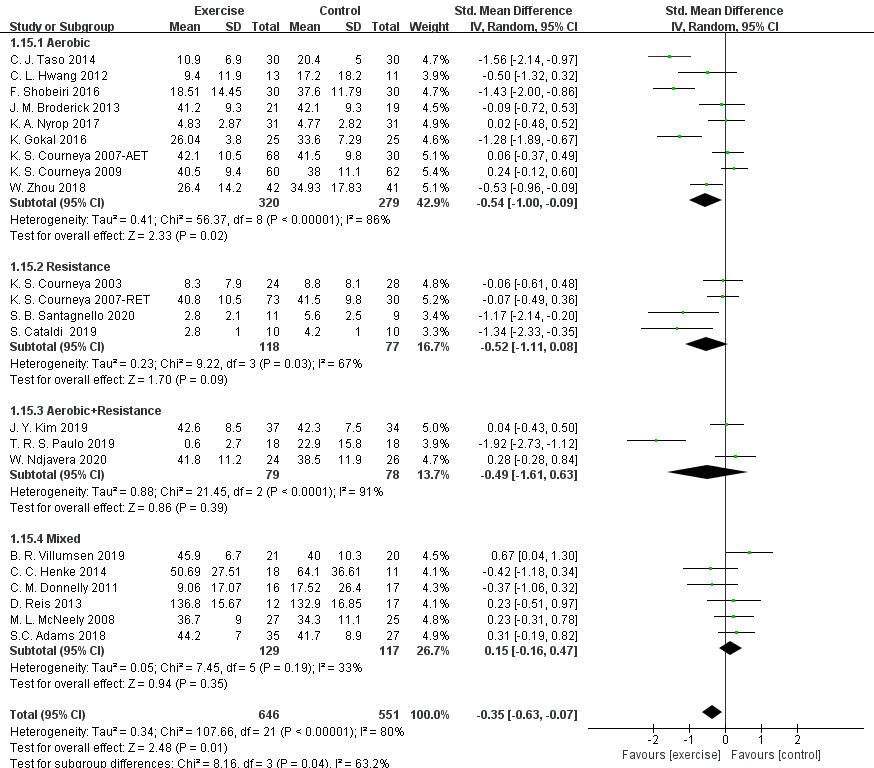


**(a)**


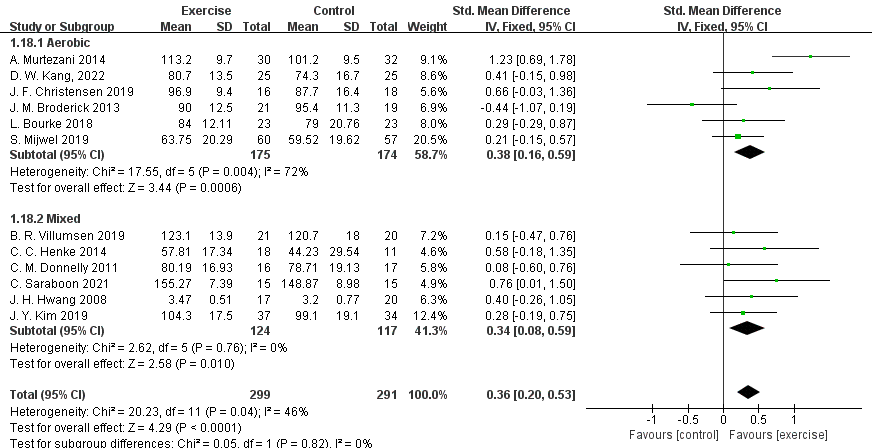


**(b)**

**Figure S2.** Forest plots of the effects of the type of exercise intervention on (a) cancer-related fatigue and (b) quality of life, AET = aerobic exercise training, RET = resistance exercise training.


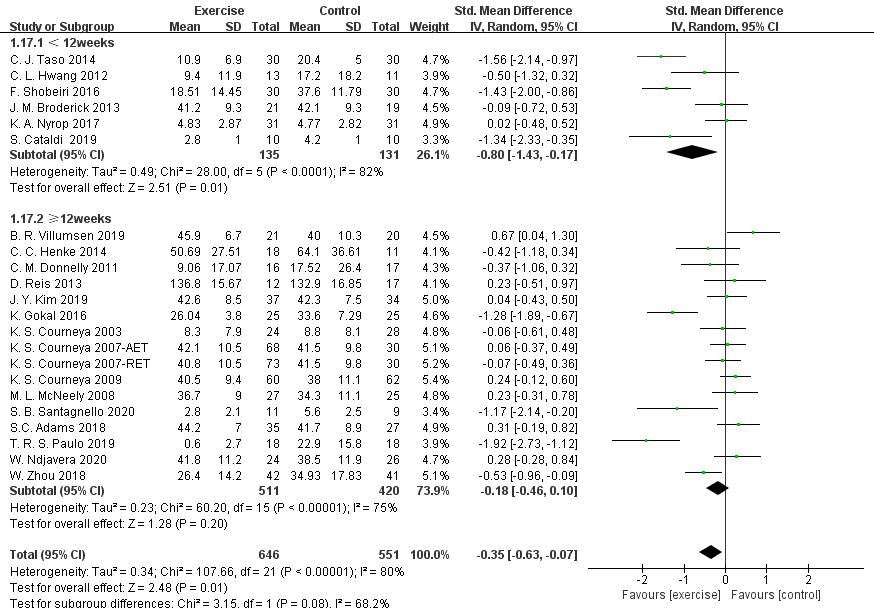


**(a)**


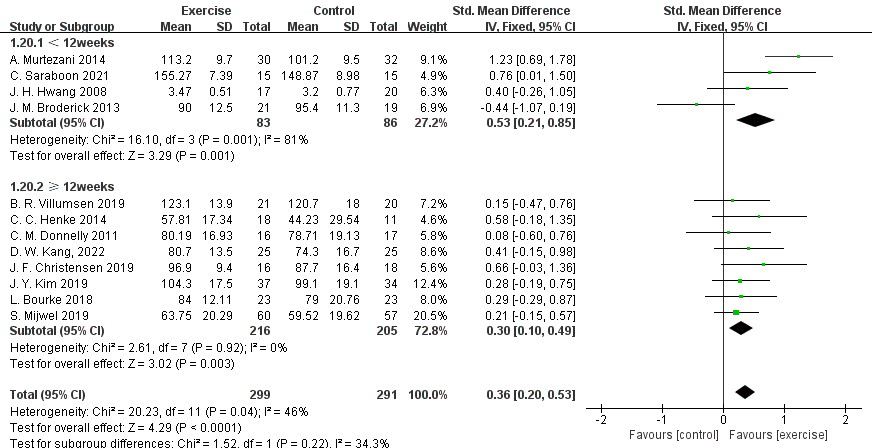


**(b)**

**Figure S3.** Forest plots of the effects of the duration of exercise intervention on (a) cancer-related fatigue and (b) quality of life, AET = aerobic exercise training, RET = resistance exercise training.


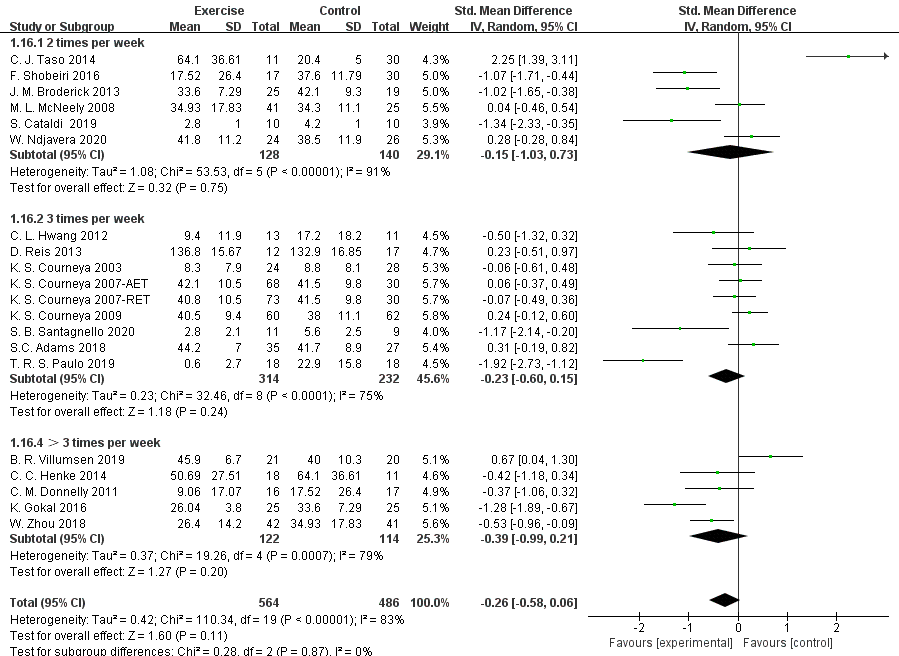


**(a)**


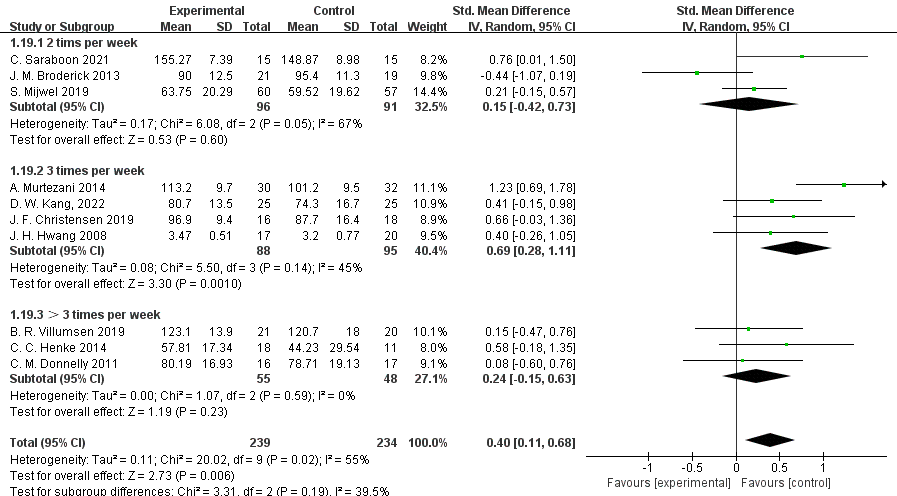


**(b)**

**Figure S4.** Forest plots of the effects of the frequency of exercise intervention on (a) cancer-related fatigue and (b) quality of life, AET = aerobic exercise training, RET = resistance exercise training.


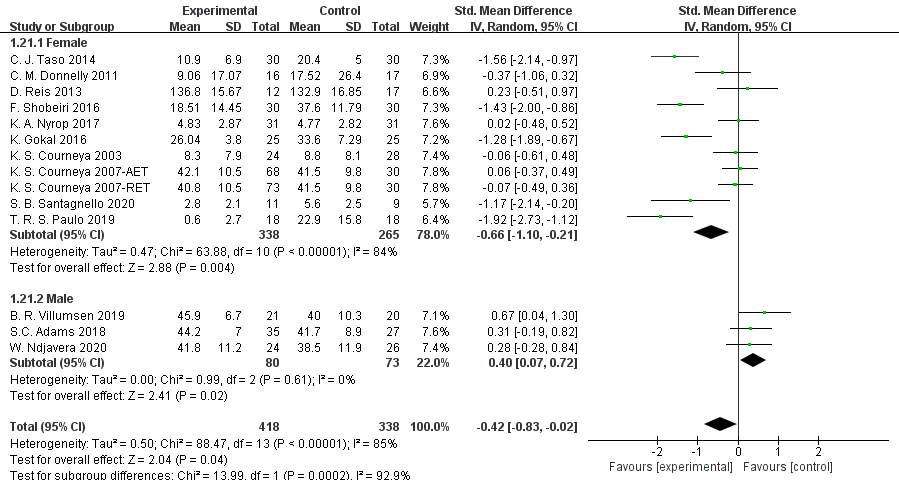


**(a)**


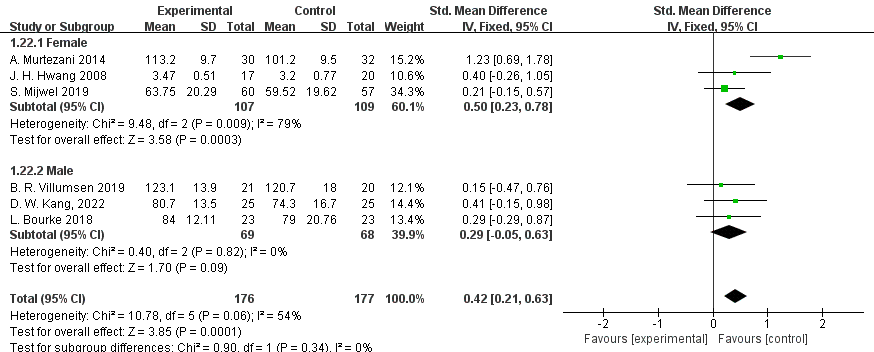


**(b)**

**Figure S5.** Forest plots of the effects of the gender of cancer patients on (a) cancer-related fatigue and (b) quality of life, AET = aerobic exercise training, RET = resistance exercise training.
